# *In situ* lattice tuning of quasi-single-crystal surfaces for continuous electrochemical modulation†

**DOI:** 10.1039/d2sc01868c

**Published:** 2022-05-19

**Authors:** Biao-Feng Zeng, Jun-Ying Wei, Xia-Guang Zhang, Qing-Man Liang, Shu Hu, Gan Wang, Zhi-Chao Lei, Shi-Qiang Zhao, He-Wei Zhang, Jia Shi, Wenjing Hong, Zhong-Qun Tian, Yang Yang

**Affiliations:** State Key Laboratory of Physical Chemistry of Solid Surfaces, Pen-Tung Sah Institute of Micro-Nano Science and Technology, College of Chemistry and Chemical Engineering, IKKEM, Xiamen University Xiamen 361005 China yangyang@xmu.edu.cn

## Abstract

The ability to control the atomic-level structure of a solid represents a straightforward strategy for fabricating high-performance catalysts and semiconductor materials. Herein we explore the capability of the mechanically controllable surface strain method in adjusting the surface structure of a gold film. Underpotential deposition measurements provide a quantitative and ultrasensitive approach for monitoring the evolution of surface structures. The electrochemical activities of the quasi-single-crystalline gold films are enhanced productively by controlling the surface tension, resulting in a more positive potential for copper deposition. Our method provides an effective way to tune the atom arrangement of solid surfaces with sub-angstrom precision and to achieve a reduction in power consumption, which has vast applications in electrocatalysis, molecular electronics, and materials science.

## Abstract 2

We reported a new method capable of adjusting the lattice structure of solid surfaces with sub-angstrom precision and achieved *in situ* and continuous control over electrochemical activity.

## Graphical abstract

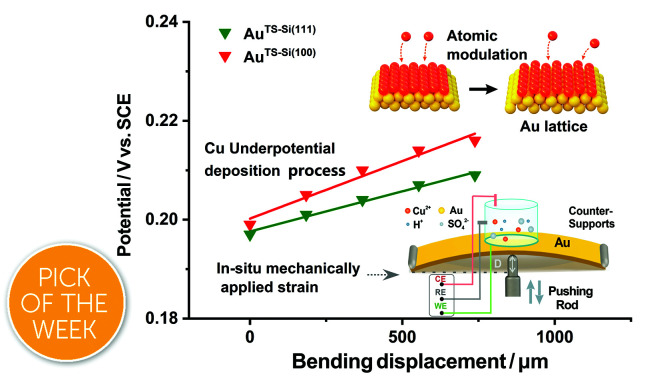

## Introduction

Attempts to prepare solid materials with well-defined structures have gathered tremendous interest in physics,^1^ chemistry,^2,3^ materials science,^4–6^ and the semiconductor industry.^7,8^ For a metal, a variety of properties depend critically on its crystal structure,^9^ including electricity,^10–12^ transparency,^13^ and catalysis.^14,15^ In the past decades, various methods have been developed to fabricate metal crystals with distinct structures, such as liquid-phase crystal growth,^16,17^ programmed annealing,^18^ and epitaxial film deposition.^19^ When a metal serves as an electrocatalyst, a small modulation in its surface crystal structure will create substantial changes in its contact with adsorbed molecules.^20,21^ It is often thought that this feature, in turn, offers an opportunity to improve the performance of electrocatalysts.^22–24^ Nevertheless, the *in situ* modulation of the surface structure with atomic precision remains a challenge in the engineering of metallic crystals.^25,26^

To modulate the crystal structure, approaches based on atomic force microscopy (AFM)^27,28^ or tip-enhanced photoluminescence (TEPL) spectroscopy^29^ have been suggested and realized in several materials recently, for instance, the method of bending the sample using a tip^30^ that changes the crystal structure of the imposed area. The ability to tune the crystal structure *in situ* creates the opportunity to study a number of fundamental processes like the evolution of the piezoelectric effect. However, the AFM-based method is time-consuming, and the active area of the sample is small.^31^ A mechanically controllable break junction (MCBJ) provides an alternative approach to modulating the surface strain of metal.^32–34^ Owing to the unique three-pivot architecture, there is a reduction ratio as large as 3 to 5 orders of magnitude between the displacement of the underneath actuator and the movement of the atoms on the metallic sample.^35–37^ Consequently, the MCBJ can control the distance between two adjacent atoms of the sample with a sub-angstrom precision,^38–40^ which is more refined to apply strain to the metal surface than previous methods.^41,42^ Since the atomic arrangement of the metal surface determines the crystal structure of a metal film, the MCBJ thus can tune the crystal structure with atomic precision. Underpotential deposition (UPD) has been a hotspot in both electrocatalysis and the integrated circuit industry in recent years.^43,44^ It can fabricate atomic metallic layers with lower power consumption and thus has promising applications in the synthesis of highly active nanostructured catalysts and a series of processes used for manufacturing integrated circuits. Furthermore, the UPD process is sensitive to the minor change in surficial atom arrangement, which provides a characterization tool to investigate the evolution of the crystal structure.^45^

Herein, we develop a versatile method for *in situ* and continuously modulating surface structures of the quasi-single-crystal metal film inspired by the MCBJ technique, named mechanically controllable surface strain (MCSS). We selected an Au film as the prototypical sample because it is the most extensively used substrate in electrocatalysis and surface science. Two Au films with different initial strains were fabricated by using a template stripping method and were placed onto a homebuilt MCSS setup equipped with a three-electrode electrochemical module. When Cu atoms were electrochemically reduced onto the surface of the Au films, clear UPD peaks emerged at different potentials which served as an indicator for the electrochemical activity of the Au film. With this upgraded break junction technique, we modulated the surface structure of Au films at the atomic scale and improved their electrochemical activities as indicated by the positive shifts of UPD peaks. The mechanically controlled atomic arrangement, as well as the improved electrochemical activities, were supported by AFM characterization and reproduced by density functional theory (DFT) simulations.

## Results and discussion

### Modulation of the crystal surface with MCSS

**Fig. 1:**
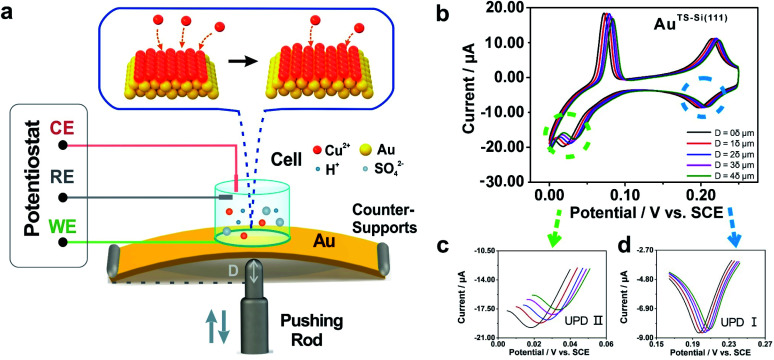
Illustration of the lattice strains induced in a quasi-single-crystal Au film. (a) Schematic diagram of the homebuilt strain modulation system. (b) Cyclic voltammograms of the Au^TS-Si(111)^ film taken in 1 mM CuSO_4_ and 50 mM H_2_SO_4_ aqueous solution were obtained before and after loading stresses. The insets represent the varying bending displacement of the film, which was utilized to modulate the strain of the film. Enlargements of the reductive peaks that appeared in (c) the first step and (d) the second step of the Cu UPD process. The scan rate of the potential was 10 mV s^−1^.

To modulate the surficial atom arrangement of metals, we made some modifications to a conventional MCBJ setup and finally assembled an MCSS setup. The MCSS setup is sketched in Fig. 1a. A quasi-single-crystal Au film was fixed onto the homebuilt MCSS setup, with a micro electrolytic cell placed on it and a stepping motor acting as the underneath pushing rod. The quasi-single-crystal Au films were prepared using the Si/SiO_2_-templated stripping strategy,^46^ as detailed in Fig. S1.† Notably, preparing reliable electrode surfaces is essential for the fabrication of mechanically stable molecular devices, and the electrochemical activity is closely related to the structure of the gold surface.^47^ Thus, the natural oxide layer was removed from the surface of Si wafers before the Au deposition process for directly modulating the Au surface by the structural difference of Si single crystals.^48–50^ By employing Si(111) and Si(100) wafers as templates, we prepared two types of Au films with exposed (111) planes but different initial interatomic spacings, labeled as Au^TS-Si(111)^ and Au^TS-Si(100)^ films, where “TS” denotes “template stripping” and they represent that the Au films were deposited on Si single-crystal wafers with respective crystal planes. During the electrochemical characterization, the Au film, Pt wire, and Pt black wire served as the working electrode (WE), the counter electrode (CE), and the reference electrode (RE), respectively. The Pt black wire is utilized as the RE rather than the common saturated calomel electrode to avoid the adsorption of the chloride ion on the Au surface. The preparation and characterization of Pt black wire are given in Fig. S2 and S3.†

For the electrochemical studies, the Au films were supported on the elastic polymethyl methacrylate (PMMA) substrate. We then assembled a mechanical modulation system containing components of the film sample, three-pivot configuration, and electrochemical module. Fig. S4† shows the photos of the assembled system. We put conductive stickers on both the tip of the stepping motor and the bottom of the PMMA substrate. To start, the underneath stepping motor was moved upward at a low velocity. Once the tip of the stepping motor was brought into contact with the bottom of the PMMA substrate, the movement of the stepping motor was halted by using an electrical feedback circuit. The displacement of the stepping motor in such a case was flagged as zero. To apply strain for the Au film, the underneath stepping motor was moved further to bend the PMMA substrate. For a stepping motor, the actual displacement of each step might be a little deviated from the nominal value when it is loaded. Before the bending experiments, as shown in Fig. S5,† we had calibrated the displacement of our stepping motor under the same loading conditions. We found that the precise displacement of our stepping motor with one step is 9.23 nm.

Due to the mechanism of MCSS, the underneath stepping motor induced strains across the sample surface, giving rise to controllable variation of the lattice spacing in the surficial atoms. A parameter named reduction ratio, *r*, is utilized to describe the control precision by referring to the conventional MCBJ method. In particular, *r* is derived from the ratio between the change in the lattice spacing and the displacement of the stepping motor. In our MCSS setup, the reduction ratio *r* reached about 1.5 × 10^−4^ in the center point of the sample surface (ESI, Section S5†).^51,52^ Thus, one step of the stepping motor, *i.e.*, a 9.23 nm displacement, gave rise to a 1.38 pm change in surficial interatomic spacing. Because the center of the surface witnessed the maximum value of *r* and a smaller *r* leads to more precise control, the above derived 1.38 pm value shows the capability of our setup in tuning the lattice spacing of quasi-single-crystal surfaces with picometer precision.

### Electrochemical characterization of Au^TS-Si(111)^ and Au^TS-Si(100)^ films

The electrochemical component allows the *in situ* investigation of the evolution of electrochemical activity along with strain, which in turn gives an indicator for identifying the controllable atomic adjustment of the surface crystal structure. To compare the electrochemical activity of quasi-single-crystal materials in the presence and absence of strain, we employed the UPD of Cu at the as-fabricated Au film as the prototypical reaction. UPD is of general interest in electrochemistry because it provides an ideal model system for the study of the electrode–electrolyte interface.^53^ In our work, it was selected owing to its sensitivity towards the atomic arrangements of a solid surface. Fig. 1b shows the cyclic voltammograms of our Au^TS-Si(111)^ film that were measured from +0.300 to 0 V in an aqueous solution containing Cu^2+^ cations. The bulk deposition of copper ions on the gold surface occurred after 0 V (Fig. S7A†). Notably, two other pairs of redox peaks emerged that correspond to two sequential adsorption/desorption processes. To be concise, we denote the reductive peak at high potential as Cu UPD I and the peak at low potential as Cu UPD II (Fig. 1b). A significant feature of the UPD II peak is that it splits into two, which is extensively utilized as an indicator for quasi-single-crystal Au with high quality.^54,55^

We then applied strains onto the Au film by using the MCSS system. As shown in Fig. 1b, we froze the stepping motor at different displacements and performed the CV measurements. For convenience, we defined a stroke of 2 × 10^4^ steps as displacement *δ*, and in the subsequent experiments, we set the movement of the stepping motor to be 1*δ*, 2*δ*, 3*δ*, and 4*δ* to generate different strains in the Au films. Because the occurrence of bulk deposition would damage the ordered structure of the Au^TS-Si(111)^ surface, the potential was swept from +0.3 V to 0 V to avoid the Cu bulk deposition. This sweeping window ensures the reproducibility of UPD I and UPD II in the consecutive CV measurements (Fig. S7B†), which indicated that the ordered structure of the Au^TS-Si(111)^ film was well preserved. Fig. 1c shows the evolution of Cu UPD II along with the bending displacement of the Au^TS-Si(111)^ film, while Fig. 1d shows that of Cu UPD I. For both UPD I and UPD II, it is observed that the peaks shift in a positive direction. This is important because it shows that the consumed energy to trigger the deposition of Cu atoms can be reduced by increasing the strain of an Au film. In addition, we performed reversible experiments (Fig. S8†) and found that the positions of the Cu UPD peaks slightly shifted negative but did not coincide with the values in the bending process when we decreased the bending displacement. This result implied that a partial plastic deformation was induced in the bending process, possibly due to the existence of plastic deformation defects.

The results shown in Fig. 1b and c demonstrate that the Cu UPD process is significantly affected by the applied strains. It is well known that the Cu UPD process occurs in the inner layer of the double electric layer. Thus, it is sensitive to the tuning of the Au surface structure, which agrees with our experimental findings. In contrast, the outer-sphere electron transfer process that occurs in the outer Helmholtz plane will not expect a strain effect. To verify this hypothesis, we measured the redox reactions of potassium ferricyanide and hexaammineruthenium(iii) chloride, which are well-validated outer-sphere redox systems in electrochemistry. As shown in Fig. S9 and S10,† the redox peaks of potassium ferricyanide and hexaammineruthenium(iii) chloride are located at the same potential while applying different strains, which further confirms that the positive shift of Cu UPD potentials is attributed to the modulation of the Au surface.

**Fig. 2:**
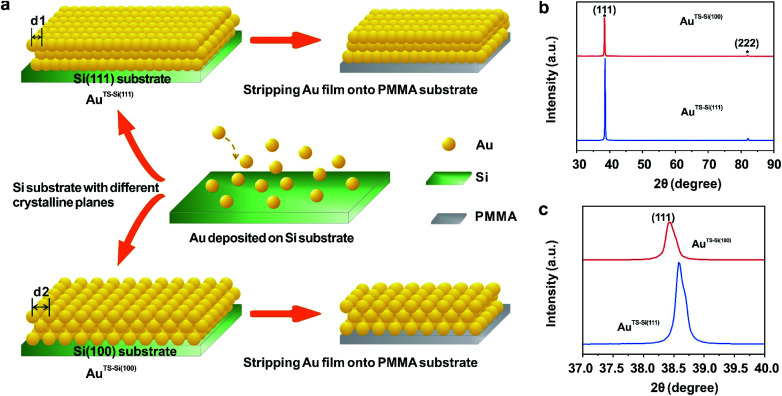
The characterization of the initial states of prepared Au films. (a) Schematic preparation of two types of Au films. Gold was first deposited on two types of Si single-crystal wafers, including Si(111) and Si(100), which were named Au^TS-Si(111)^ and Au^TS-Si(100)^, respectively. The Au film obtained from the Si(100) template has larger atomic spacing than that obtained from the Si(111) template (*d*_2_ > *d*_1_). Then the deposited Au films were stripped onto PMMA substrates for electrochemical characterization. (b) The XRD spectra of two prepared Au films. The two peaks marked by asterisks are assigned to Bragg reflections from the Au(111) and Au(222) crystallographic planes. (c) The enlarged XRD patterns of the Au(111) peaks for the two Au films. The peak shifts of Au(111) show there is an increase in lattice spacing for the Au^TS-Si(100)^ film compared to the Au^TS-Si(111)^ film.

**Fig. 3:**
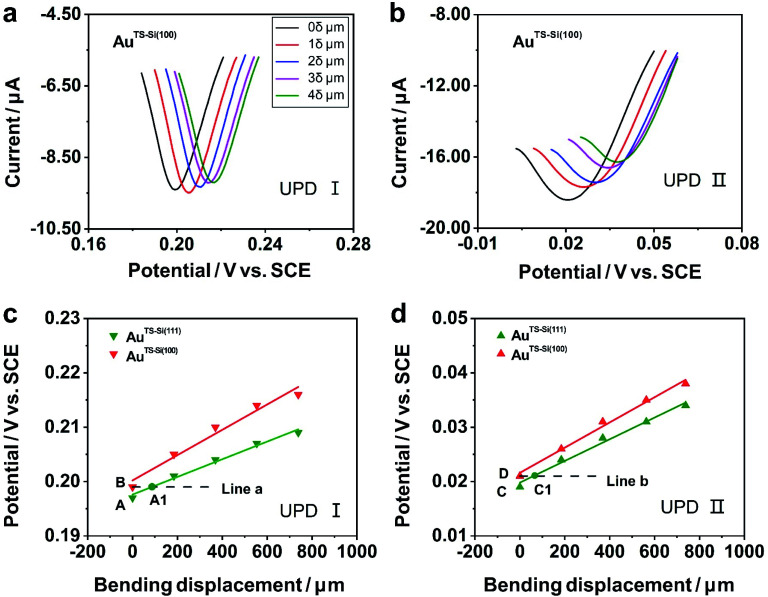
Cyclic voltammograms of Cu UPD on quasi-single-crystal Au^TS-Si(100)^ films under different strain conditions. (a) Cu UPD I on the Au^TS-Si(100)^ film; (b) Cu UPD II on the Au^TS-Si(100)^ film in an aqueous solution containing 1 mM CuSO_4_ and 50 mM H_2_SO_4_. The scan rate of the potential was 10 mV s^−1^. The inset in (a) represents the varying bending displacement of the film, which was utilized to modulate the strain of the film. Evolution of the underpotential of (c) Cu UPD I and (d) Cu UPD II, along with bending displacement of the Au film. In particular, green for the Au^TS-Si(111)^ film, and red for the Au^TS-Si(100)^ film. The bottom dashed lines (line a and line b) represent the contour plot of the electrochemical activity for Cu UPD I and Cu UPD II of the Au^TS-Si(100)^ film without bending. A1 and C1 are points where line a intersects the green line and line b intersects the green line, indicating the electrochemical activities for Cu UPD I and Cu UPD II of the Au^TS-Si(111)^ film are equal to that of an unbending Au^TS-Si(100)^ one at such bending displacements.

To explore the general capability of our MCSS method in modulating the electrochemical activity, we carried out the Cu UPD studies on the samples of Au^TS-Si(100)^ films. As shown in the schematic of Fig. 2a, the Au^TS-Si(100)^ film was prepared by using a Si/SiO_2_-templated stripping strategy, where the gold was deposited on the Si(100) single-crystal wafers. The results of the X-ray diffraction (XRD) experiments showed that both the Au^TS-Si(111)^ film and Au^TS-Si(100)^ film were 〈111〉 oriented, as shown in Fig. 2b.^56,57^ However, the XRD peak of Au(111) facets corresponding to Au^TS-Si(100)^ and Au^TS-Si(111)^ shifted from 38.64° to 38.42° (Fig. 2c), indicating an increase in lattice spacing for Au^TS-Si(100)^ in comparison with Au^TS-Si(111)^.^58,59^ For the Au^TS-Si(100)^ film, the underpotential of Cu UPD I and Cu UPD II without bending is 0.199 V and 0.021 V, respectively (Fig. S11†), which are more positive than those of Cu UPD I and Cu UPD II on Au^TS-Si(111)^ films (0.197 V and 0.019 V). The comparison between the two samples shows that the electrochemical activity of Cu UPD is sensitive to the surface lattice strain, and the surface with larger atomic spacing, as induced by lattice strain, has enhanced electrochemical activity in the Cu UPD process. In addition, we performed the CV measurements for Au^TS-Si(100)^ films under different strain conditions in an aqueous solution containing Cu^2+^ cations. The whole CV curves measured for Au^TS-Si(100)^ are given in Fig. S11,† while the enlargement of the regions involved with the Cu UPD I and Cu UPD II is given in Fig. 3a and b. In both figures, the UPD peaks shift in a positive direction, which is consistent with that observed for the Au^TS-Si(111)^.

To study the mechanism of the electrochemical activity modulations by the modified MCSS method, we compared the evolution of the UPD peaks quantitively. Fig. 3c and d plot the potentials of Cu UPD I and Cu UPD II *versus* the bending displacement. The linear fitting of the data points represented the capability of continuously modulating electrochemical activity in our experiments. In particular, we plotted two auxiliary lines parallel to the *X*-axis, labeled as line a and line b in Fig. 3c and d, respectively. These lines indicated that, under the bending conditions imposed by our MCSS method, the Au^TS-Si(111)^ film can reach an electrochemical activity the same as the unbending Au^TS-Si(100)^ film. We found the electrochemical activities of the Au^TS-Si(111)^ film were equal to those of Au^TS-Si(100)^ without bending at the bending displacement of *X*_A1_ = 87.96 μm for Cu UPD I, and at *X*_C1_ = 67.26 μm for Cu UPD II, respectively. The values of bending displacement obtained from the UPD II process are less than the values of the UPD I process (Fig. 3c and d). It is rational because both the UPD I and UPD II peaks are contributed by the underpotential deposition of Cu atoms on the surface of gold layers with single-crystal structures, while the UPD I process corresponds to the structural transition from (√3 × √3)*R*30° adlayers and the UPD II process represents the formation of the full Cu monolayer.^53,54,60^ Because the UPD I process and the UPD II process represent the deposition of Cu ions in different regions on the surface of gold with a single-crystal structure, our result indicated the homogeneous variation of a gold single-crystal surface in a region of applied strain. The strain at grain boundaries was not further studied in our experiment since the electrochemical activity of the Cu UPD process has little correlation with the increase of grain boundary density as reported in previous work.^61,62^ It is thus verified that the enhanced electrochemical activity of the bent Au film, as indicated by the UPD process, is contributed by strain engineering on the quasi-single-crystal surface. This finding showed that, mechanical bending provides a simple yet feasible means of improving electrochemical activity for metal, indicating vast applications in electrochemistry.

### Electrochemical characterization of the roughened Au^TS-Si(111)^ film

We further explored the mechanism of the enhanced electrochemical activity of the Au film by our MCSS method. During the bending experiment, there might be two kinds of microscopic evolution that occurred to the Au film. For one thing, the bending operation might generate irregular atom islands. Previous studies showed that the carbon monoxide catalytic oxidation of an electrode would improve when irregular atom islands were generated on it,^63^ because the atom islands give rise to a large amount of highly active dislocations, such as steps, terraces, and defects.^64^ For the other thing, the bending experiment could adjust the interatomic spacing and transform the film sample from a low active single-crystal surface to a more active one. We will demonstrate below that it is the latter one that contributed to the enhanced activity in our experiment.

**Fig. 4:**
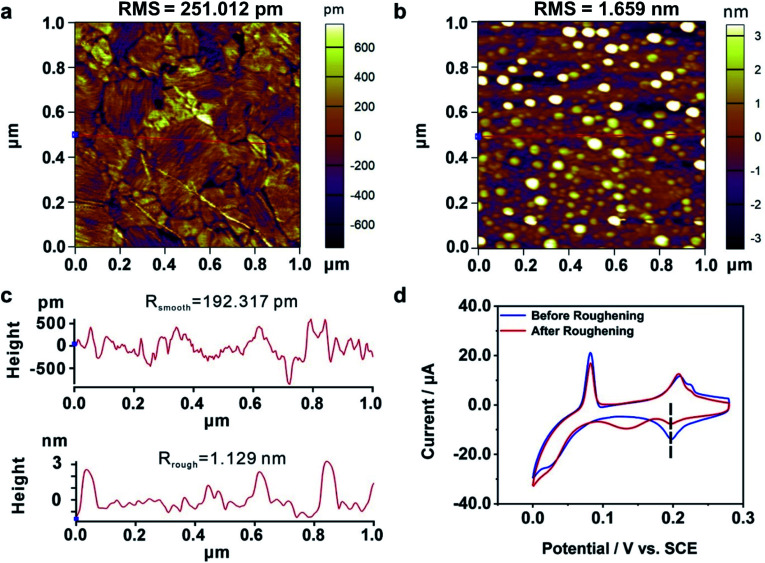
The electrochemical activity of a roughened Au film. AFM images of an Au film (a) before and (b) after the roughening process. The RMS roughnesses of the surfaces (total area = 1.00 μm^2^) are as follows: Au film without the roughening process, 251.012 pm; Au film with the roughening process, 1.659 nm. The bright white dots in (b) represent those generated Au atom islands. (c) The roughness measurements for the Au film before (upper panel) and after (bottom panel) the roughening process. The red lines in (a) and (b) represent the corresponding measurement areas. (d) Cyclic voltammograms of Cu UPD on an Au film before (blue) and after (red) the roughening treatment.

We carried out a control experiment on a deliberately prepared Au film where a large number of Au atom islands were loaded. For this purpose, an Au film was roughened by employing an electrochemical roughening method called the oxidation and reduction cycle (ORC) method.^65,66^ In particular, the Au film was repeatedly oxidized and reduced when the applied electrode potential followed a tailored potential–time function. The ORC procedure was repeated 5 times to generate a large amount of Au islands. Fig. 4a and b give the AFM characterization of an Au film before and after the roughening process, showing that lots of atom islands were generated. The root mean square (RMS) roughness of the Au dramatically increased from 0.25 nm to 1.66 nm by the roughening process. In addition, we plotted two straight lines (Fig. 4a and b, red lines) and calculated the average roughness, as shown in Fig. 4c. The average roughness is slightly less than RMS, but the order of magnitude remains the same, showing excellent uniformity. Fig. 4d shows the CV measurements carried out on an Au film before and after the roughening treatment. It is observed that the UPD I peak splits into two, *i.e.*, a peak is located at the same potential as the untreated Au film, and a new peak emerged at a more negative potential. These findings show that the generated Au atom islands reduced the exposed area of (111) planes, leading to a smaller quantity of Cu atoms that deposited at the characteristic UPD I potential. Moreover, there is no peak observed at the more positive potentials, indicating that the generated Au atom islands are unable to reduce the minimum bias for triggering the UPD process. Therefore, it is the adjustment of the interatomic spacing of the surficial crystal plane other than the increased roughness that contributed to the enhanced electrochemical activity of our bent Au film samples.

### DFT calculation for bonding energies of Cu atoms on the Au film

**Fig. 5:**
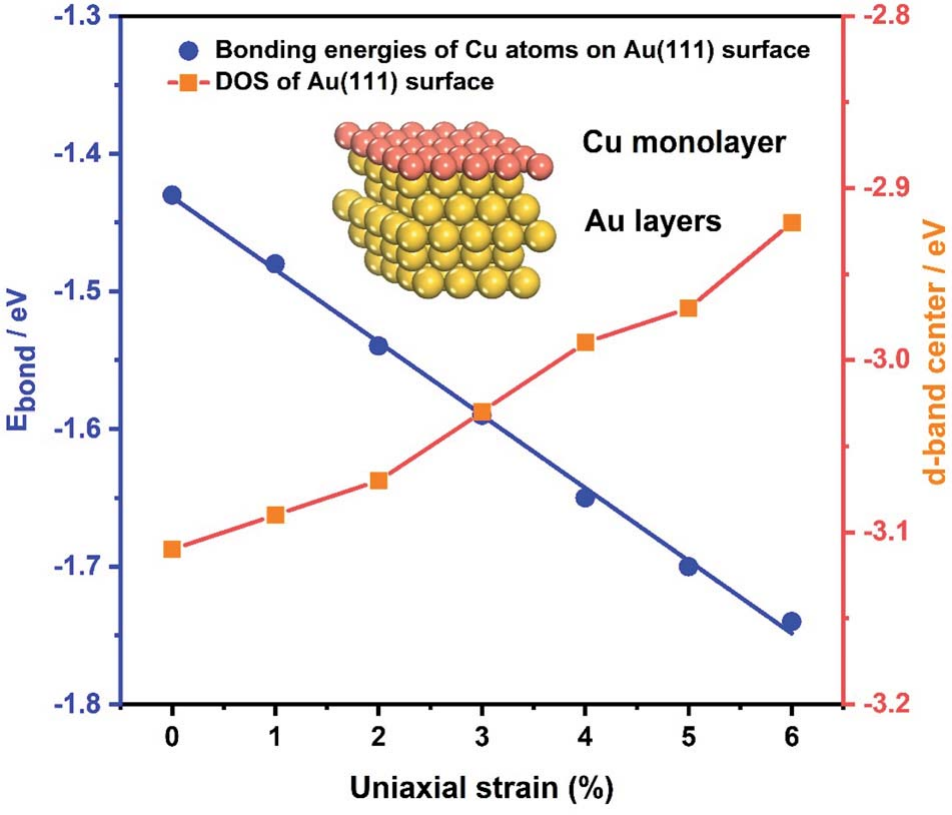
Theoretical calculations. The blue axis on the left shows the bonding energies of Cu atoms and the Au(111) surface as a function of the uniaxial strain. The line is linear and fits the data points. The orange axis on the right shows the calculated DOS of Au(111) under the applied tensile strains. Inset: schematic of the Cu monolayer deposited on the Au(111) layers.

As the samples are bent by our MCSS setup, there is a change in the interatomic spacing of the surficial crystal plane, which gives rise to a strain for the Au film. To further identify the role of strain in enhancing the electrochemical activities of the Au film, the bonding energies of Cu ions UPD on the Au(111) film under a series of strain conditions were calculated by DFT calculations. We applied a tensile strain to the surface of the Au(111) layers, increasing the strain from 0% to 6% by a step of 1%. Fig. 5 (blue line) displays the bonding energy of Cu atoms and Au(111) surfaces under different strain conditions. For unstrained Au(111), the value of bonding energy is −1.43 eV, indicating that the adsorption of the Cu monolayer on the Au(111) surface is a thermodynamic spontaneous process, corresponding to the Cu UPD process in the electrochemical experiments. It is apparent that the bonding energies of Cu atoms and the Au(111) surface increase with the applied tensile strain of an increasing lattice constant (Fig. 5). This result leads to the positive shift of deposition potential of Cu atoms on the Au(111) surface, which is consistent with that observed in our experiments. Meanwhile, the bonding energies of Cu atoms on the Au(111) surface under compressive strain are reduced with the compressive strain (Fig. S12†). Significantly, the bonding energies are approximately linear to the applied strain, which means the continuous modulation of the UPD activity by the applied strain.

In addition, the effect of applied strain on the bonding energies of Cu atoms on the Au(111) surface is strongly related to the Au 5d band. Previous theoretical studies reported that the surface reactivity of the strained metal surfaces increases with lattice expansion, which is accompanied by the up-shift of the metal d states.^67,68^ Thus we calculated the d electron density of states (DOS) of the Au(111) layers under a series of applied tensile strains, as shown in Fig. 5 and S13.† For tensile strain in the range of 0% to 6%, the d-band center is gradually up-shifted and close to the Fermi energy level (*E*_F_), resulting in enhanced surface activity and facilitating the bond interaction with the Cu atoms. Surface reactivity increases with lattice expansion, following a concurrent up-shift of the metal d states. The d-band upshifts under the applied tensile strains, showing a qualitative consistency with the decreased bonding energies of Cu atoms on the Au(111) surface. Such results further support the effect of strain on the improved activity toward the Cu UPD process.

## Conclusions

In conclusion, we developed a novel method capable of modulating the interatomic spacing of metal surfaces and realized *in situ* control of their electrochemical activities. The method, named MCSS, not only enables us to modulate and monitor the changes in the quasi-single-crystal surface through electrochemical characterization but also retains the sub-angstrom precision of MCBJ in tuning the surficial interatomic spacing. We conducted copper deposition on the prototypical samples of Au quasi-single-crystal films and utilized the UPD peaks to study their electrochemical activities. Enhanced electrochemical activity was observed as two distinct UPD peaks shifted positively with the upward movement of the pushing rod. The results implied that the MCSS method can modulate the electrochemical activity *in situ* and continuously rather than discretely. In addition, the electrochemical activity was demonstrated to be enhanced by the lattice strain on the metal surface both experimentally and theoretically. The MCSS method allows researchers to mechanically tailor the chemical and physical properties of a solid surface under ambient conditions, and thus provides a versatile platform for a variety of research in catalysis, materials science, solid-state physics, and molecular electronics.

## Data availability

The data supporting the findings of this study are available within the article and in the ESI.†

## Author contributions

Y. Y. conceived the original concept, supervised the project, and wrote the first version of this manuscript. B. F. Z., J. Y. W., and Q. M. L. performed the experiments. X. G. Z. conducted the theoretical calculations. S. H., Z. C. L., G. W., and S. Q. Z. contributed to the optimization of the MCSS setup and electrodes. J. S. and W. H. compiled the autoencoders. B. F. Z., Z. Q. T. and Y. Y. analysed the data and revised the manuscript. All authors discussed the results and approved the final version of the manuscript.

## Conflicts of interest

There are no conflicts to declare.

## Supplementary Material

SC-013-D2SC01868C-s001
