# Supplementary material for: In situ lattice tuning of quasi-single-crystal surfaces for continuous electrochemical modulation
Source: Chem Sci. 2022 May 19;13(26):7765–72. doi: 10.1039/d2sc01868c (PMC9258404; doi:10.1039/d2sc01868c)
Supplement: SC-013-D2SC01868C-s001 [file SC-013-D2SC01868C-s001.pdf]

Electronic Supplementary Material (ESI) for Chemical Science.
This journal is © The Royal Society of Chemistry 2022

Supporting Information

In-situ lattice tuning of quasi-single-crystal surfaces for continuous electrochemical modulation

Biao-Feng Zeng,[‡a] Jun-Ying Wei,[‡a] Xia-Guang Zhang,[‡a] Qing-Man Liang,[a] Shu Hu,[a] Gan Wang,[a] Zhi-Chao Lei,[a] Shi-Qiang Zhao,[a] He-Wei Zhang,[a] Jia Shi,[a] Wenjing Hong,[a] Zhong-Qun Tian[a] and Yang Yang[*a]

[a.] State Key Laboratory of Physical Chemistry of Solid Surfaces, Pen-Tung Sah Institute of Micro-Nano Science and Technology, College of Chemistry and Chemical Engineering, IKKEM, Xiamen University, Xiamen 361005, China

[‡.] These authors contributed equally

[*.] Correspondence: yangyang@xmu.edu.cn (Y. Y)

Table of Contents

Section S1. Experimental Materials and Methods
Section S2. Preparation of Electrodes
Section S2.1. Preparation of Working Electrode
Section S2.2. Preparation of Platinum Black Wire
Section S3. Design and Construction of Experimental Devices
Section S4. Determined the Distance of One Step of the Pushing Rod
Section S5. Calculation of the Attenuation Ratio of the MCSS Setup
Section S6. Determination of Potential Scanning Range
Section S7. The Reversible Experiments for $Au^{TS\text{-}Si(111)}$
Section S8. The Electrochemical Measurement of Potassium Ferricyanide and Hexaammineruthenium(III) Chloride
Section S9. The Electrochemical Measurement of $Au^{TS\text{-}Si(100)}$
Section S10. The Calculation of Bonding Energies for Copper Ions Deposited on Au Surfaces
Supplemental References

Experimental Section

Section S1. Experimental Materials and Methods

Cyclic voltammogram (CV). The electrolyte solution consists of 1 mM $CuSO_4$ and 50 mM H_2SO_4. A platinum black wire served as the reference electrode. The potential difference between it and the conventional saturated calomel electrode is 0.7 V. During the CV measurement, the potential was swept in a range of 0.30 V to 0.00 V at a scan rate of 10 mV/s.

Chloroplatinic acid (H_2PtCl_6), lead acetate ($(CH_3COO)_2Pb$), Sulfuric acid (H_2SO_4), Copper(II) sulfate pentahydrate ($CuSO_4 \cdot 5H_2O$), potassium chloride (KCl), Potassium Ferricyanide ($K_3[Fe(CN)_6]$) and hexaammineruthenium(III) chloride ($[Ru(NH_3)_6]Cl_3$) were purchased as the analytical grade from Sinopharm Chemical Reagent Co., Ltd. and used without further purification. All solutions were prepared by using fresh Milli-Q water with a resistivity of 18.2 MΩ·cm.

X-ray powder diffraction (XRD) experiments were carried out by employing a Rigaku Ultima IV diffractometer with Cu Kα radiation (λ = 1.54056 Å). Data was collected between 2θ angles of 30° and 90° at a scan rate of 10° min^{-1}.

Section S2. Preparation of electrodes

Section S2.1. Preparation of Working Electrode

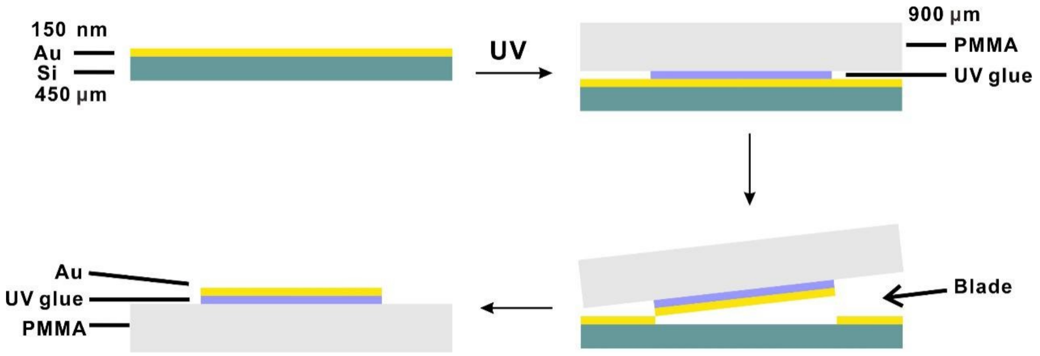

Figure S1. Schematic diagram of the fabrication of quasi-single-crystal Au films via Si/SiO_2-templated stripping strategy.

To fabricate Au quasi-single-crystal films as working electrodes, we stripped the Au films, $Au^{TS\text{-}Si(111)}$ and $Au^{TS\text{-}Si(100)}$, from the surfaces of Si(111) and Si(100), then further used them to test the CVs date at different strain conditions.

Before Au film deposition, the natural oxide layer on the surface of the crystalline Si wafer has been etched as follows: the 4-inch wafer was first cleaned using the piranha solution (H_2SO_4 : H_2O_2 = 3 : 1, v/v) at 250 °C for 15 min. Then the Si wafer was rinsed with the deionized water several times till the piranha solution is completely removed. The surface of the Si wafer was subsequently etched in dilute hydrogen fluoride solution for 10 s. Finally, the Si wafer was rinsed using deionized water three times and dried with nitrogen gas. After cleaning the silicon wafers, a 150 nm Au thin layer was deposited on the single-crystal Si wafers, including Si(111) and Si(100) by using E-beam Evaporation. Semenza. G. et al. reported that the films with lower root mean square roughness could be obtained by mechanical or chemical stripping.[1] Based on these ideas, we formed the PMMA/Au/Si(h,k,l) structure by attaching polymethyl methacrylate to the ultraviolet (UV) adhesive. Then, the gold/silicon surface was stripped as the working electrode, as shown in Figure S1. Two platinum wires with 99.5% purity were purchased from Jiaming Platinum Nonferrous Metals Co., Ltd. One of the platinum wires was used as the counter electrode, and another one was used as the reference electrode by depositing platinum black.

Section 2.2. Preparation of Platinum Black Wire

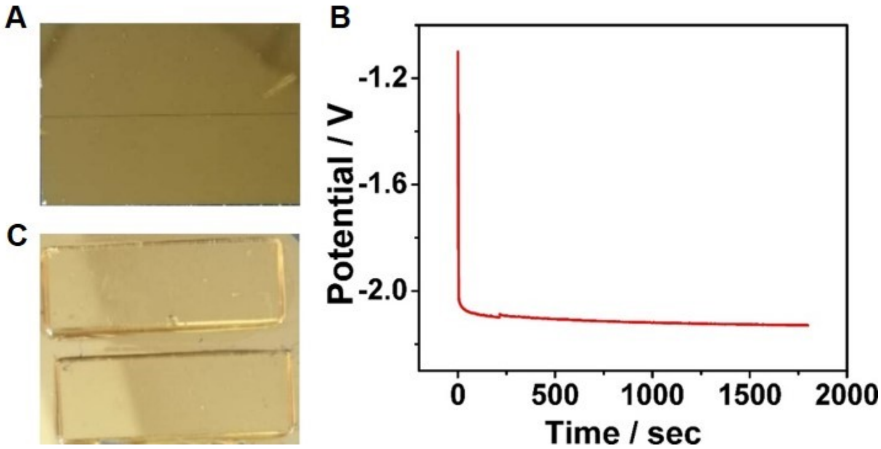

Figure S2. Two kinds of working electrodes and the preparation of platinum black wire. (A) Two pieces of 30×10 mm Si(111)/Au after cutting. (B) A potential-time diagram of platinum black deposited on a platinum wire. (C) Two pieces of PMMA adhered to strip the inner gold surface with UV glue.

The commercial saturated calomel electrode, AgCl electrode, etc. as reference electrode will introduce Cl^- into the electrolyte solution,[2] which will cause pollution to the experimental system and affect the quality of the experiment. To avoid Cl^- contamination, we used platinum black wire as the reference electrode. A platinum black wire with a constant current of 1500 s at a current of 2 mA was prepared by pre-treating the platinum wire in a plating solution consisting of 5 mM chloroplatinic acid and 0.3 mM lead acetate. The data of chronoamperometry is shown in Figure S2B.

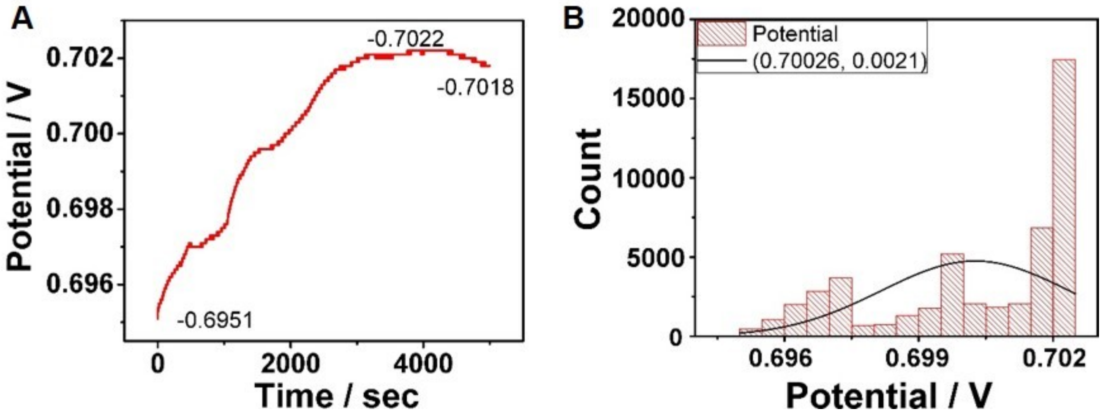

Figure S3. The electrode stability of platinum black wire. (A) Open circuit potential-time diagram between platinum black wire and calomel. (B) Statistical analysis of the potential data in (A) to obtain a potential range.

To confirm the electrode stability of self-made platinum black wire, we tested the difference of open circuit potential between the platinum black wire and saturated calomel electrode in 1 mM $CuSO_4$ plating solution as time goes by, as shown in Figure S3A. Various range of potential is between -0.6951 V and -0.7022 V within 5000 s and the maximum potential drift value is 0.0071 V, which could be negligible. We performed a statistical analysis on the potential of Figure S3A and found that the average potential difference between the platinum black wire and the saturated calomel electrode is about -0.70 V, as shown in Figure S3B.

Section S3. Design and Construction of Experimental Devices

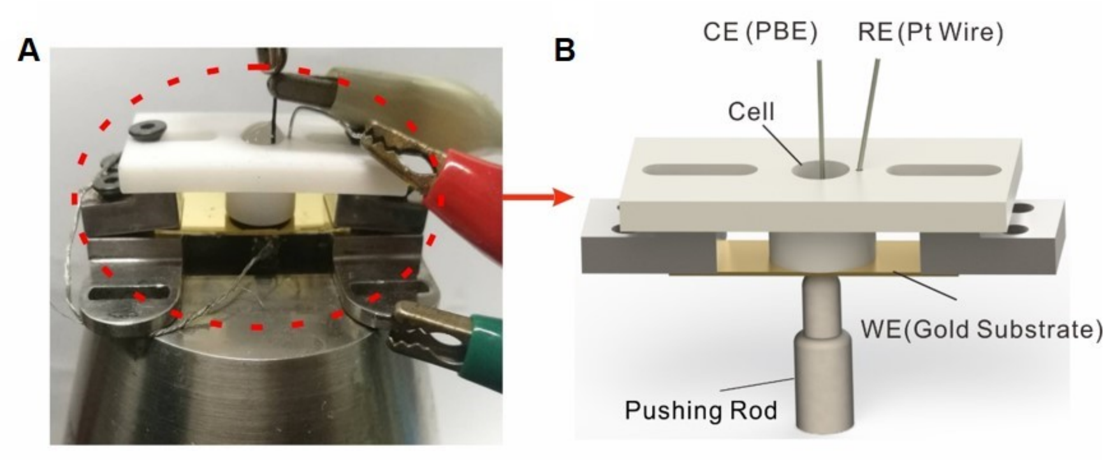

Figure S4. Photograph and schematic diagram of the experimental device. (A) Photograph of the experimental setup. (B) Magnification of the red circle portion of (A).

The experimental device combined the two characteristics of precise regulation of strain and *in-situ* electrochemical measurement. Thus, the surface strain on the metal substrate could be precisely controlled and measured under different stresses. Figure S4 shows the photo and schematic diagram of the experimental setup. The pushing rod in Figure S4B is Linear Actuator, which could control motor steps more accurately by adding an autoencoder. A digital multimeter was employed to control the movement of the pushing rod to just touch the lower surface of the substrate and controlled the strain displacement by the number of motor steps to change the bending gold-plated substrate. In addition, two conductive stickers were put on both the tip of the stepping motor and the bottom of the PMMA to access a feedback system.

Section S4. Determined the Distance of One Step of the Pushing Rod

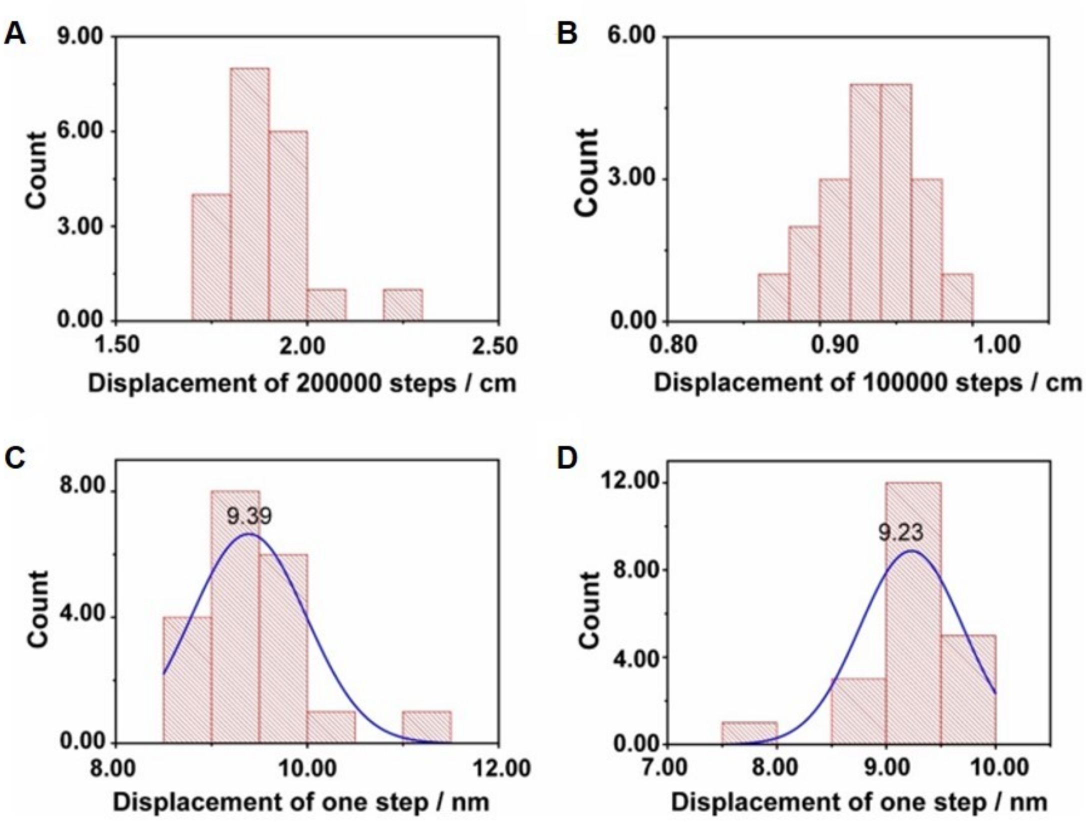

Figure S5. Calculated the relationship between the moving distance of the pushing rod and the number of steps. (A) The displacement of the pushing rod moves 200000 steps without the substrate. (B) The displacement of the pushing rod motor moves 100000 steps when a substrate is PMMA. (C) The displacement of the pushing rod moves one step without the substrate. (D) The displacement of the motor moves one step when a substrate is PMMA.

We measured the displacement of the pushing rod without and with the PMMA substrate on the top by performing 20 tests in parallel, as illustrated in Figure S5A and Figure S5B. In the meanwhile, Figure S5C shows that the average displacement of one step is 9.39 nm without load. However, due to the reaction force of load, stepping displacement would be reduced to 9.23 nm when the PMMA/Au substrate is loaded during the whole experiment process, as illustrated in Figure S5D.

Typically, we used the surface of Au film as a working electrode and defined δ= 184.6 μm as the displacement of 20000 steps.

Section S5. Calculation of the Reduction Ratio of the MCSS Setup

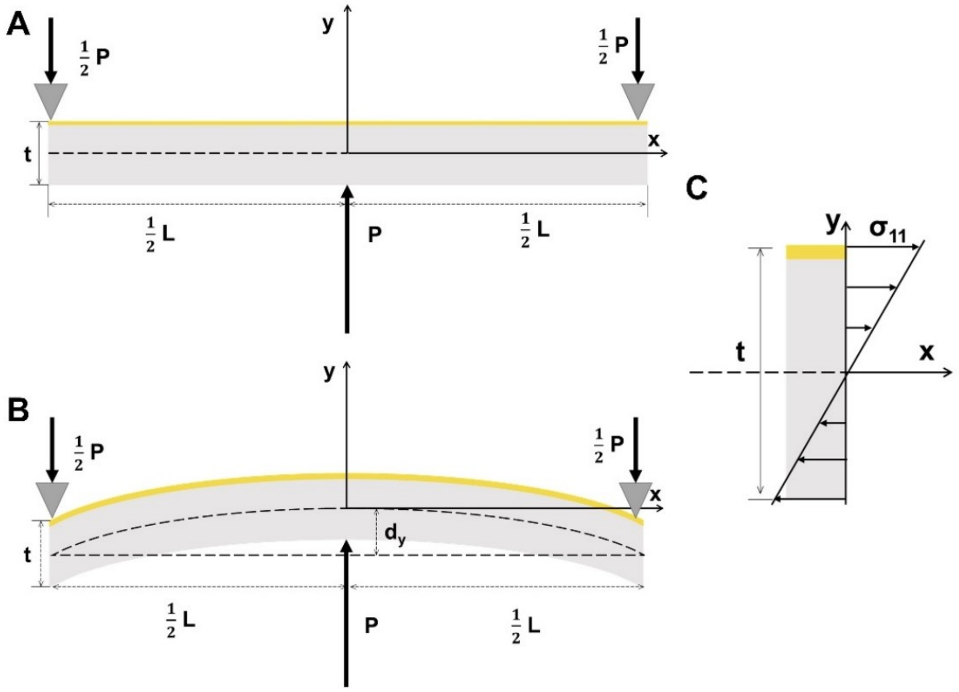

Figure S6. Schematic of the three-pivot architecture and model for attenuation ratio calculation. (A) The area between two counter supports of the sample is modeled as a slender beam of length L and thickness t, the pushing rod exerts force P in the center of the beam. The force of endpoints x = $\pm 0.5L$ is 0.5P. (B) The beam is bent relative to the neutral axis y = 0 by the displacement d_y. (C) Linear normal stress (σ_{11}) and associated strain (ε_{11}) distribution across the cross-section are induced by the bending moment.

According to the previous literature,[3] the reduction ratio ***r*** of MCSS can be calculated by the following formula, $r = u1/u2 = 6tx/L^2$, where $u1$ is the spacing increase of metal film surface, $u2$ is the movement distance of the pushing rod, t is the substrate thickness, x is the horizontal distance from the center of the pushing rod, and L is the distance between the two counter supports (Figure S6). In our modified MCSS setup, t=1 mm is the thickness of the PMMA substrate, while the thickness of deposited Au film is ignored since its thickness is several orders of magnitude smaller than the PMMA substrate. The beam length L is 20 mm. Thus, within a 10 μm of the center of the sample, the reduction ratio $r = \frac{6 \times 10^{-3} \times 10^{-6}}{(2 \times 10^{-2})^2} = 1.5 \times 10^{-4}$. In fact, the calculated attenuation ratio is only applicable to the tiny region in the middle of the

sample. In the experiment, the force required to apply strain to the sample is larger than the calculated value due to the influence of the non-central region.

Section S6. Determination of Potential Scanning Range

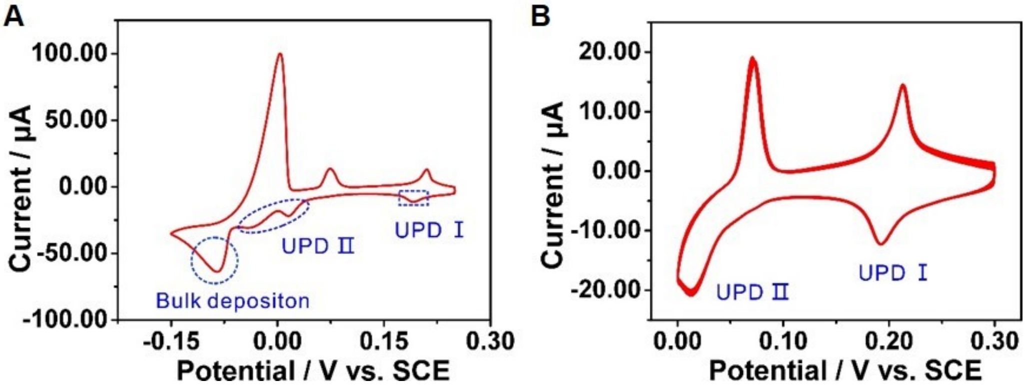

Figure S7. Cyclic voltammograms of Cu deposition on the Au surface. (A) CVs of Cu deposition on the Au surface measured from +0.30 V to -0.10 V. (B) CVs of Cu UPD measured from +0.30 V to 0.00 V by using platinum black wire as a reference electrode. The potentials of UPD peaks did not shift after 20 consecutive scanning cycles. The potential of UPD I was 0.197 V, and the potential of UPD II was 0.013 V.

We employed the as-prepared Au film as the working electrode for electrochemical cleaning in 50 mM sulfuric acid and the results are shown in Figure S7A. CV measurements were performed by replacing the 50 mM H_2SO_4 solution in the electrolytic cell with a mixed electrolyte of 1 mM $CuSO_4$ and 50 mM H_2SO_4. The bulk deposition of copper ions on the gold surface occurred after 0.00 V. Thus, the potential scan lower limit was set at 0.00 V and the scan range of Cu UPD was from 0.30 V to 0.00 V by using a platinum black wire as the reference electrode, as shown in Figure S7B. The test result is consistent with the previous literature reports.[4]

Section S7. The Reversible Experiments for $Au^{TS\text{-}Si(111)}$

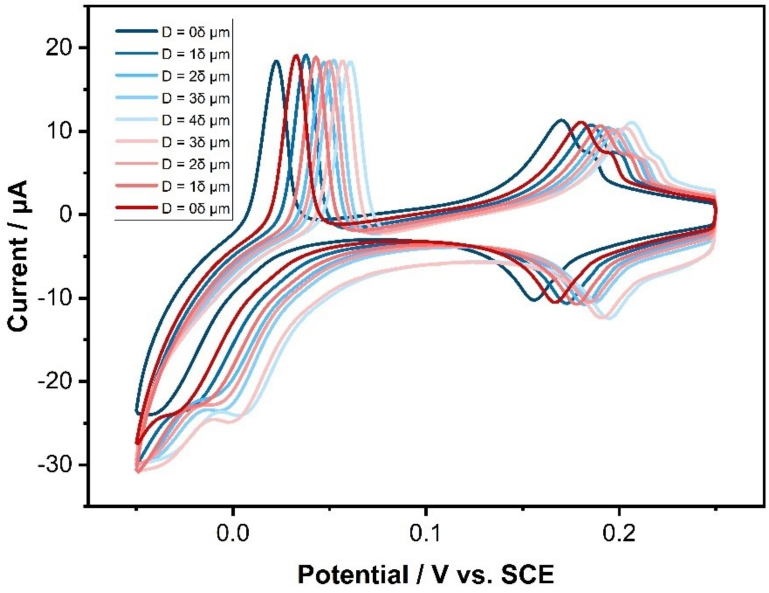

Figure S8. Cyclic voltammograms of a reversible experiment for $Au^{TS\text{-}Si(111)}$. Blue lines: the CVs measured in Au film with increasing bending displacement; red lines: the CVs measured in Au film with decreasing bending displacement. The electrochemical measurements were taken in 1 mM $CuSO_4$ and 50 mM H_2SO_4 at a scan rate of 10 mV/s. RE: platinum black wire. CE: platinum wire.

Section S8. The Electrochemical Measurement of Potassium Ferricyanide Hexaammineruthenium(III) Chloride

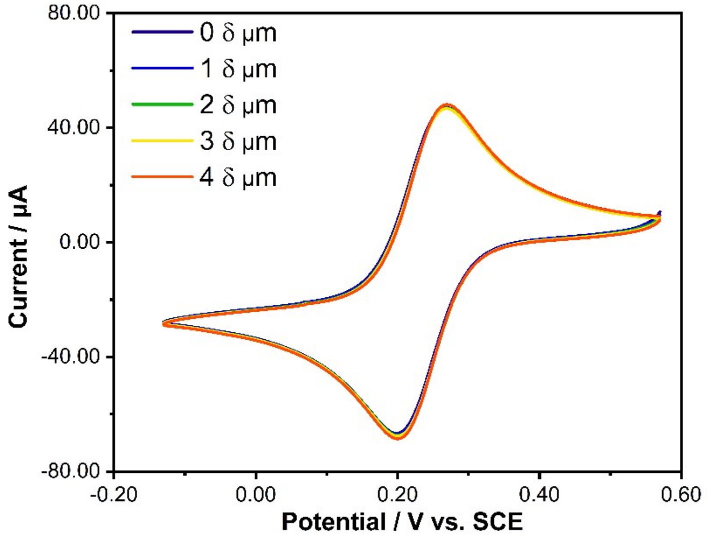

Figure S9. Cyclic voltammograms of $Au^{TS\text{-}Si(111)}$ film in potassium ferricyanide solution under five strain conditions. The CVs of the $Au^{TS\text{-}Si(111)}$ surface were taken in 1 mM $K_3[Fe(CN)_6]$ and 0.5 M KCl at a scan rate of 10 mV/s.

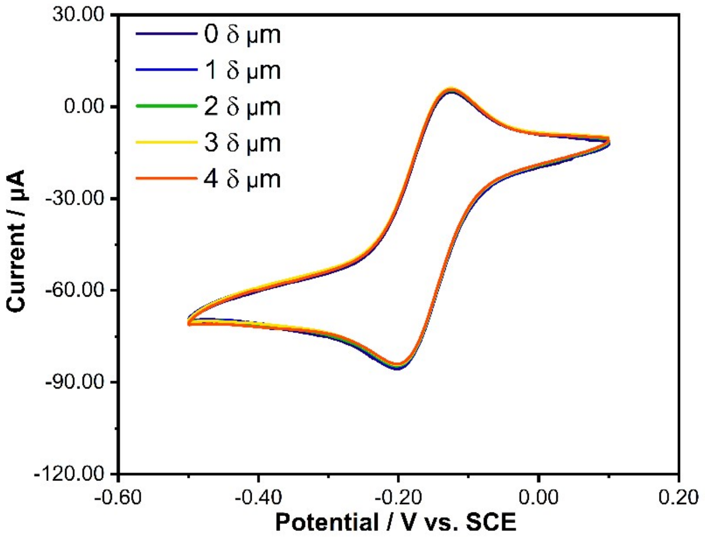

Figure S10. Cyclic voltammograms of $Au^{TS\text{-}Si(111)}$ film in hexaammineruthenium(III) chloride solution under five strain conditions. The CVs of an $Au^{TS\text{-}Si(111)}$ surface were taken in 1 mM $[Ru(NH_3)_6]Cl_3$ and 0.1 M KCl at a scan rate of 10 mV/s.

We carried out a control experiment to study the influence of the modulation of Au surface structure on the outer-sphere electron transfer process. Specifically, we measured the redox

reaction of potassium ferricyanide in the electrolyte solution, which consists of 1 mM $K_3[Fe(CN)_6]$ and 0.5 M KCl. The Au electrode was cleaned and activated in the solution of 0.5 M H_2SO_4 before cyclic voltammogram measurement. During the electrochemical measurement, the Au electrode served as the working electrode, the Pt wire served as the counter electrode and a Pt black wire served as the reference electrode. The potential was swept at a scan rate of 10 mV/s. The results of CVs are shown in Figure S9. There is no shift in redox potential for potassium ferricyanide under different applied strains. Also, we measured the $[Ru(NH_3)_6]^{3+}$ and $[Ru(NH_3)_6]^{2+}$ electrochemical redox couple in aqueous solutions containing KCl as the supporting electrolyte. The result is shown in figure S10. There is no shift in redox potential for hexaammineruthenium(III) chloride under different applied strains.

Section S9. The Electrochemical Measurement of $Au^{TS\text{-}Si(100)}$

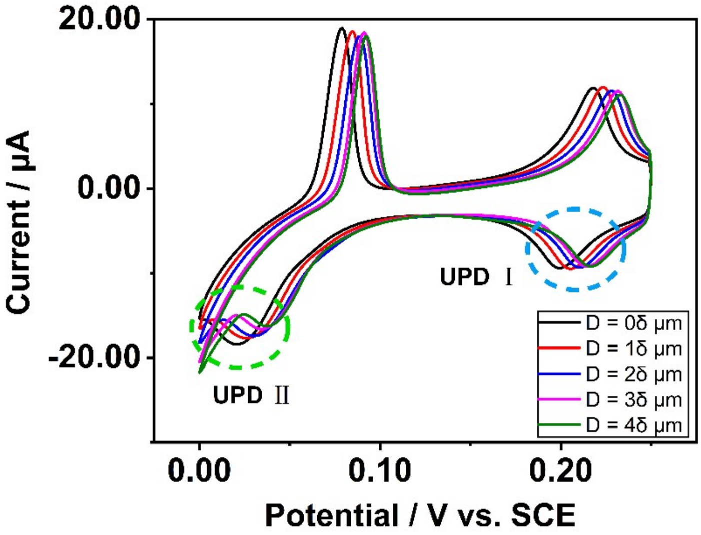

Figure S11. Cyclic voltammograms of $Au^{TS\text{-}Si(100)}$ film under five strain conditions. The CVs of an $Au^{TS\text{-}Si(100)}$ surface were taken in 1 mM $CuSO_4$ and 50 mM H_2SO_4 at a scan rate of 10 mV/s. RE: platinum black wire. CE: platinum wire.

We prepared gold films stripped from Si(100) which was defined as $Au^{TS\text{-}Si(100)}$ and tested CVs date under five strain conditions as illustrated in Figure S11. The blue and green dotted circles shown in Figure S11 are corresponding to UPD I and UPD II.

Section S10. The Calculation of Bonding Energies for Copper Ions Deposited on Au Surfaces

All DFT calculations were performed by using the Perdew-Burk-Ernzerhof (PBE) functional of generalized gradient approximation (GGA)[5] in Vienna ab initio simulation package (VASP),[6] in which projector augmented potential (PAW) method[7] was applied to describe the electron-ion interactions. The Beck-Jonson damping (DFT-D3) method was employed to correct the van der Walls interactions of the system. An energy cut-off of 450 eV was used for the wave functions and energies were converged to 10^{-6} eV, and the force was converted to 0.01. The Methfessel-Paxton method with a broadening factor of 0.1 eV was used. The Γ-centered k-point sampling grid of 12×12×12 was adopted for the primitive cell calculation, and the calculated lattice constant of Au bulk was 4.119 Å, which agreed with the experimental value of 4.078 Å. In this work, the Au bulk was simulated with a 2×2 five-layer slab, and the top three layers are relaxed, vacuum spaces of 15 Å were used to describe the surface. A Γ-centered k-point sampling grid of 6×6×1 and 18×18×1 were adopted for all single-crystal facets optimized and DOS calculation, respectively. The model of the Cu/Au surface was mimicked by a slab with five layers of Au substrate covered by a Cu monolayer, and the Cu atoms were adsorbed on the fcc site.

The bonding energies (E_{bond}) used in the work were evaluated as:

$$E_{bond} = (E_{tot} - E_{metal} - E_{nCu})/n$$

Where E_{tot}, E_{metal}, E_{nCu}, and n are the total electronic energies of Cu covered on Au surface, a clean Au slab, and surface monolayer Cu atoms, the number of surface Cu atoms, respectively.

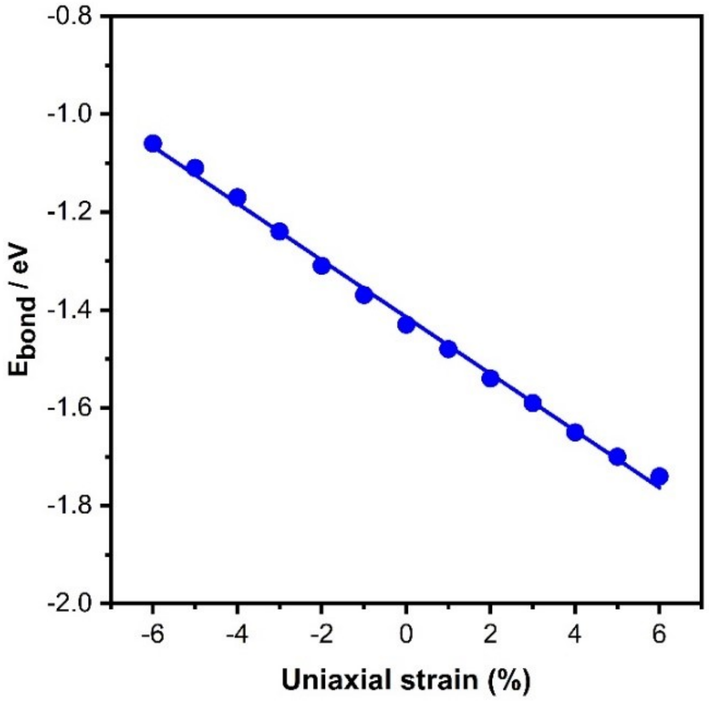

Figure S12. The bonding energies for copper ions deposited on Au(111) surfaces under a series of uniaxial strains. The negative uniaxial strain represents compressive strain and the positive uniaxial strain represents tensile strain. The line is linear and fits the data points.

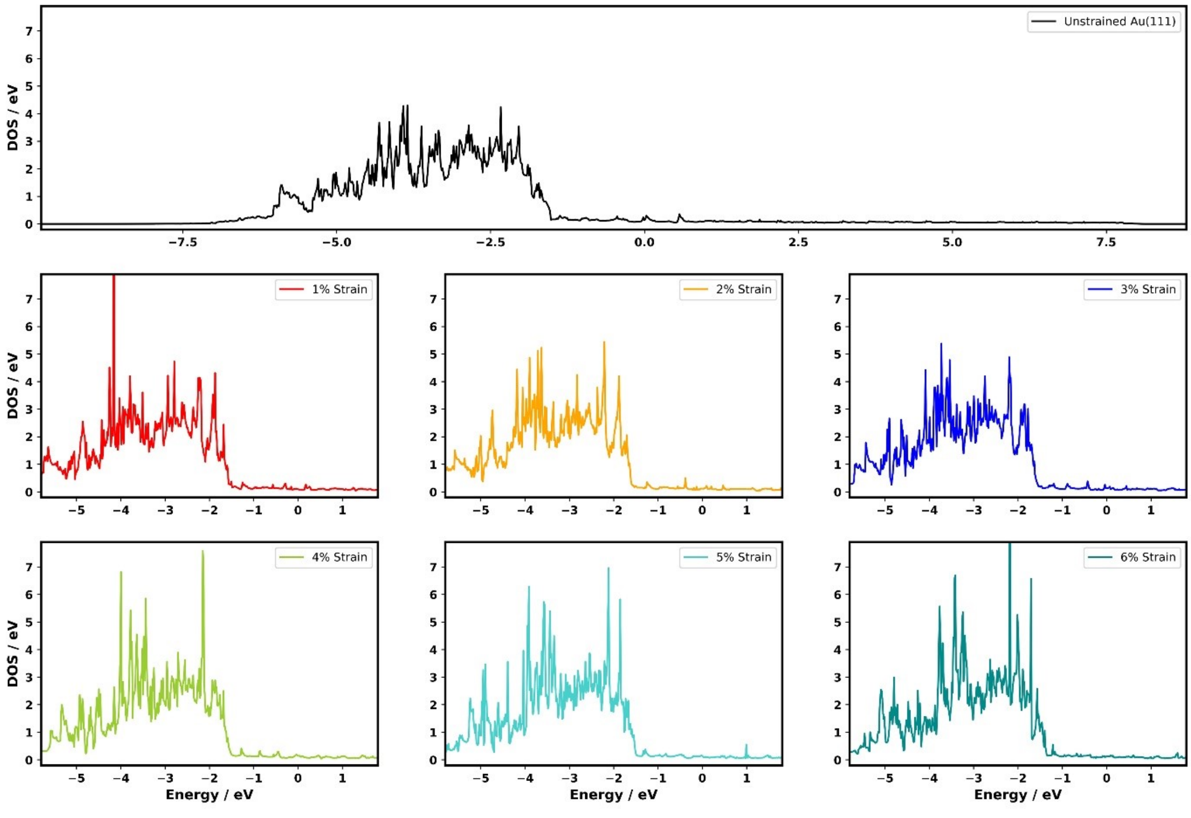

Figure S13. The calculated DOS of Au(111) surface under a series of tensile strains.

Table S1. The calculated DOS of Au(111) surfaces under a series of applied uniaxial strains.

Applied uniaxial strain / %	d-band center / eV
0	-3.11
1	-3.09
2	-3.07
3	-3.03
4	-2.99
5	-2.97
6	-2.92

Supplemental References

1. E. A. Weiss, G. K. Kaufman, J. K. Kriebel, Z. Li, R. Schalek and G. M. Whitesides, *Langmuir*, 2007, **23**, 9686-9694.
2. C. Bonnaud, I. Billard, N. Papaiconomou, E. Chainet and J. C. Lepretre, *Phys. Chem. Chem. Phys.* , 2016, **18**, 8148-8157.
3. S. A. G. Vrouwe, E. van der Giessen, S. J. van der Molen, D. Dulic, M. L. Trouwborst and B. J. van Wees, *Phys. Rev. B*, 2005, **71**, 035313.
4. E. Herrero, L. J. Buller and H. D. Abruna, *Chem. Rev.*, 2001, **101**, 1897-1930.
5. J. P. Perdew, K. Burke and M. Ernzerhof, *Phys. Rev. Lett.*, 1996, **77**, 3865-3868.
6. G. Kresse and J. Furthmuller, *Phys. Rev. B*, 1996, **54**, 11169-11186.
7. P. E. Blochl, *Phys. Rev. B*, 1994, **50**, 17953-17979.
